# Development of a code-free machine learning model for the classification of cataract surgery phases

**DOI:** 10.1038/s41598-022-06127-5

**Published:** 2022-02-14

**Authors:** Samir Touma, Fares Antaki, Renaud Duval

**Affiliations:** 1 Department of Ophthalmology, Université de Montréal, Montréal, QC Canada; 2 Centre Universitaire d’Ophtalmologie (CUO), Hôpital Maisonneuve-Rosemont, CIUSSS de L’Est-de-L’Île-de-Montréal, 5415 boulevard de l’Assomption, Montréal, QC H1T 2M4 Canada

**Keywords:** Health services, Software

## Abstract

This study assessed the performance of automated machine learning (AutoML) in classifying cataract surgery phases from surgical videos. Two ophthalmology trainees without coding experience designed a deep learning model in Google Cloud AutoML Video Classification for the classification of 10 different cataract surgery phases. We used two open-access publicly available datasets (total of 122 surgeries) for model training, validation and testing. External validation was performed on 10 surgeries issued from another dataset. The AutoML model demonstrated excellent discriminating performance, even outperforming bespoke deep learning models handcrafter by experts. The area under the precision-recall curve was 0.855. At the 0.5 confidence threshold cut-off, the overall performance metrics were as follows: sensitivity (81.0%), recall (77.1%), accuracy (96.0%) and F1 score (0.79). The per-segment metrics varied across the surgical phases: precision 66.7–100%, recall 46.2–100% and specificity 94.1–100%. Hydrodissection and phacoemulsification were the most accurately predicted phases (100 and 92.31% correct predictions, respectively). During external validation, the average precision was 54.2% (0.00–90.0%), the recall was 61.1% (0.00–100%) and specificity was 96.2% (91.0–99.0%). In conclusion, a code-free AutoML model can accurately classify cataract surgery phases from videos with an accuracy comparable or better than models developed by experts.

## Introduction

Cataract surgery is the most common surgical procedure in medicine, with an estimated 20 million surgeries performed worldwide in 2015^1^. Surgeon’s experience has been shown to influence postoperative outcomes, with higher phacoemulsification case volume associated with significantly lower complication rate and improved post-operative visual acuity^2^. One way of shortening the learning curve is by using technologies able to segment, classify and analyse surgery videos to obtain objective and data-driven feedback for skill assessment^3^. Being able to receive this feedback on a surgical performance can help trainees and surgeons identify their weaknesses and limitations. Algorithms could identify for example the accuracy or speed of certain surgical steps and compare them to a normative database and offer personalized feedback to trainees. These are concrete performance metrics than can be used by surgeons to improve their skills and follow their progress over time. A randomized controlled trial by Singh et al. showed that video-based coaching enhanced the quality of laparoscopic surgical performance^4^. While the use of AI in video analysis hasn’t yet been studied, it is reasonable to believe that a similar effect will be seen when the feedback is provided by an algorithm.

Artificial intelligence (AI) experienced great strides over the past decade in ophthalmology where multiple AI solutions have been developed^5^. Deep learning (DL), a subset of AI, uses artificial neural networks to identify intricate patterns and structure in high-dimensional datasets^6^. These networks are able to improve and fine-tune their performance based on experience. This key feature makes them applicable to many domains as powerful tools for pattern recognition and classification^7^. High-accuracy DL models have been produced for fundus photography for various pathologies However, producing such algorithms requires computer science expertise as well as substantial computing resources.

Without coding expertise, developing DL models is difficult for clinicians^8^. The recent emergence of automated machine learning (AutoML) has been a major step in the democratisation of AI. AutoML describes a set of tools and techniques for streamlining model development by automating the selection of optimal network architectures, pre-processing methods and hyperparameter optimization^9^. Using a simple graphical interface and without writing code, users can build highly-accurate machine learning (ML) models. Such models have been shown to rival hand-designed (bespoke) models^7^. Multiple studies explored the use of AutoML for the classification of medical images such as fundus photography and optical coherence tomography^7,9,10^.

To our knowledge, AutoML has not yet been applied to cataract surgery phase classification. In this study, two ophthalmology trainees without coding experience designed a DL model using AutoML in Google Cloud AutoML Video Classification to classify the phases of cataract surgery.

## Methods

### Study design and data source

Although no specific AI guidelines are available for this type of study, all efforts were made to report key terms and findings in adherence to the recently published CONSORT-AI extension^11^.

**Figure 1:**
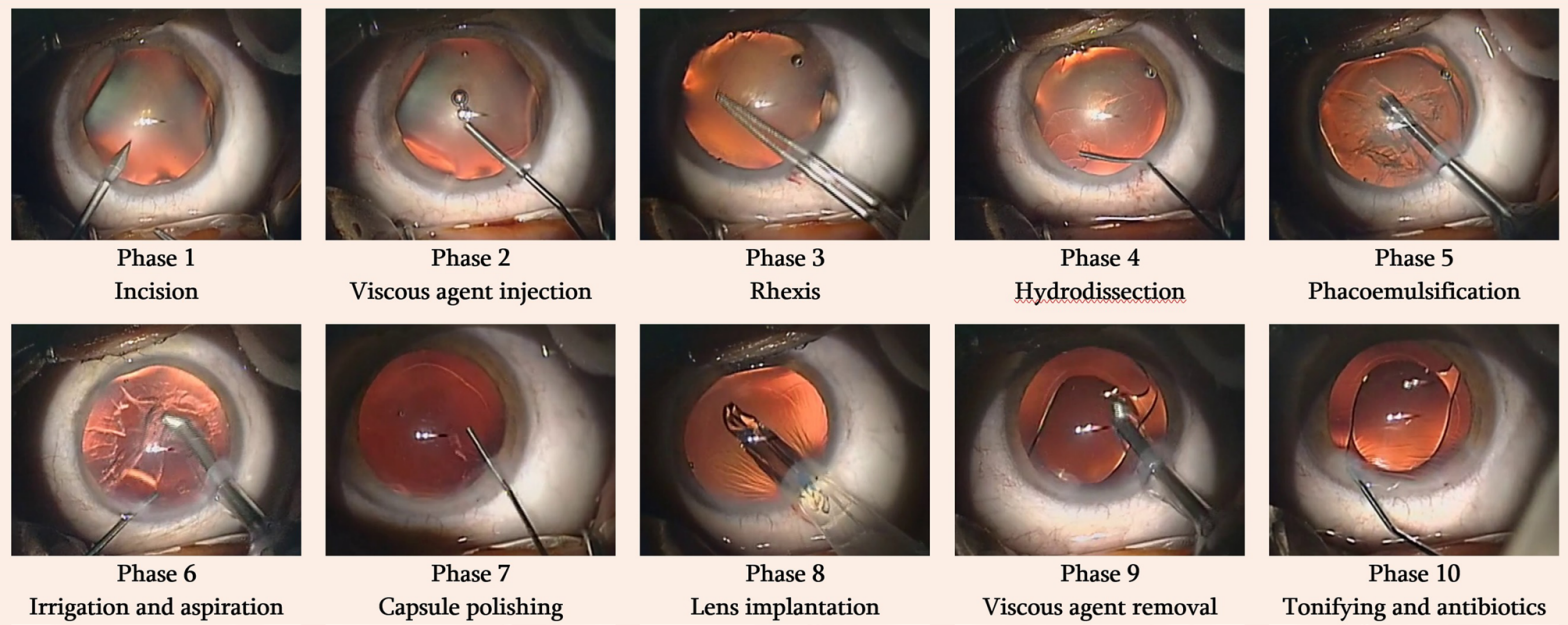
Example image for each of the ten phases of cataract surgery from the Cataract 101 dataset.

The videos used to train and test the algorithm came from the Cataract-21 and Cataract-101 datasets that were developed by the Department of Ophthalmology and Optometry of Klagenfurt University in Austria: (available at: https://klausschoeffmann.com/datasets). As stated in the metadata, surgeries were performed by ophthalmologists, including both moderately-experienced and highly-experienced surgeons. Videos were then annotated by a senior ophthalmologist into ten distinct phases: (1) incision, (2) viscous agent injection, (3) rhexis, (4) hydrodissection, (5) phacoemulsification, (6) irrigation and aspiration, (7) capsule polishing, (8) lens implantation, (9) viscous agent removal and (10) tonifying & antibiotics. Figure 1 shows representative images for each phase of the surgery. These phases are described in detail in the original article^12^. No data relabelling or re-annotation was performed.

All videos had a resolution of 720 × 540 pixels and were encoded as MP4 files, with a frame rate of 25 frames per second. The Cat-21 and Cat-101 datasets contained 21 and 101 videos respectively, for a total of 122 videos. The total duration of the videos amounted to 16 h, 26 min and 11 s. The datasets include comma-separated value (CSV) file indicating the starting and ending frame for each phase.

To perform a complete evaluation of the model, the algorithm was externally validated on a different dataset. We used 10 videos from the publicly-available CATARACTS dataset (available at https://ieee-dataport.org/open-access/cataracts). These required relabelling and reannotation as the original labels looked at tool identification. Only 9 phases could be evaluated since no capsule polishing was performed in those videos. The resolution was reduced to 720 × 540 pixels to match it with the other videos.

### Data handling and model development

Two ophthalmology trainees without coding experience (ST and FA) jointly performed the model development in Google Cloud AutoML Video Intelligence Classification. This experiment constituted a supervised learning task whereby we trained the model using labeled video segments before testing unseen video segments issued from other patients. The model does not automatically segment and assign labels to full cataract surgery videos.

The videos were uploaded in a Google Cloud bucket with CSV files indicating the video segment starting and ending time, label/phase, file path, and dataset distribution (i.e., training, or testing). We split the data into two separate sets: 90% for training and validation including hyperparameter tuning, and 10% for testing. The platform then automatically divided the training group into training (70%) and validation (30%). Each surgical video (including all the different phases) could be used either for training or testing but not both, thus respecting patient-level splits. This restriction eliminated the risk of train-test data contamination which can arise when DL models use non-clinically relevant patterns to drive predictions (e.g., image field of view, iris color, etc.). Such a problem could have resulted in overly-optimistic estimates of our model’s score.

Videos from two datasets (Cataract 101 and 21) were used to train and validate the model. In total, 1280 video segments were used, and the platform automatically uses 70% (896) for testing and 30% (384) for internal validation. In addition, 144 segments (12 surgeries) were used for testing. Supplementary table 1 shows the number of instances of each phase and the distribution across training and testing. All phases had more than 100 training videos, with a median of 114 videos per phase and ranging from 110 to 248. Regarding the testing data, the median of videos per phase was 13, ranging from 12 to 26. Viscous agent injection was the one with the highest number of instances given this phase can occur multiple times within the surgery. The external validation set included 100 video segments (10 surgeries), with 20 videos of viscous agent injection and 10 of all of the other phases (except capsule polishing which was not performed).

### Statistical analysis

Google Cloud AutoML Video Intelligence Classification provides performance metrics that are commonly used by the AI community. These include precision (positive predictive value, PPV), recall (sensitivity) as well as area under the precision/recall curve (AUPRC). AUPRC is a metric ranging from 0.5 to 1.0 with higher values indicating better discriminative performance^13^. The F1 score, which represents the harmonic mean between precision and recall, is also calculated by the platform^14^.

We extracted binary data such as true positive (TP), false positive (FP), true negative (TN) and false negative (FN) and produced contingency tables. In a multi-classification model, TP represents, for instance, the number of hydrodissection video segments that are correctly classified as hydrodissection. TN is all non-hydrodissection video segments (e.g., incision) that are not classified as hydrodissection. FP represents all non- hydrodissection segments that are inaccurately classified as hydrodissection. FN is all hydrodissection videos that are missed and classified as something else. We calculated specificity at the 0·5 threshold of using the following formula: TN/(TN + FP)^15^. To obtain an estimate of the accuracy of the model, the accuracy of each phase was manually calculated from the extracted data using the following formula: (TP + TN)/(TP + TN + FP + FN)^16^. These performance parameters were calculated to allow for a better comparison with previously published studies. Thus, we report TP, FP, TN and FN results for each phase as well as PPV, sensitivity, specificity, accuracy and F1 score. Given that this is not a screening nor a diagnostic tool, going for a threshold of 0.5 allows for a balanced trade-off between precision and recall. This is the threshold value that has been used in the other studies discussed in this paper. Finally, a confusion matrix was generated, displaying the true label and the top three misclassifications predicted by the model.

### Targeted literature search

We performed a focused literature review (MEDLINE through PubMed and Google Scholar from inception to March 3rd, 2021) to identify published DL models for cataract surgery phase classification. The flow diagram and search strategies are presented in Supplementary Fig. 1. Multiple studies have looked at cataract surgery instrument detection, but we focused on those that explored phase detection. The aim was to provide a benchmark for the discriminative performance obtained using AutoML. The goal was not to statistically compare point estimates of performance metrics and as such, we accepted differences in data handling and dataset overlap for studies that used the Cataract-21 + 101 datasets. Comparison with DL models that did not use those specific datasets was carried out to demonstrate the current state of the art.

The goal of this study was to provide an overview of the performance of AutoML for cataract surgery phase detection in comparison to bespoke DL models. The aim was not to compare accurate point estimates of performance metrics like PPV, accuracy or F1 score, and as such, we accepted differences in dataset overlap.

Our targeted literature search identified three studies describing DL models for cataract surgery phase classification^3,17,18^. All three were bespoke convolutional neural networks (CNN) that classified cataract video segments into surgical phases. Four other published studies used DL or graphical models in cataract surgery classification, but those looked at real-time phase detection where the models had to identify the surgical phase within a few seconds^19–22^. Two studies by Morita et al. also looked at cataract surgery phase detection, but the algorithm classified images (rather than videos) into specific phases (i.e., rhexis, nuclear extraction, and others)^23,24^. All the studies reported some performance metrics used in image/video classification such as PPV, accuracy or F1 score.

## Results

Overall, the AutoML model had excellent discriminative performance with an AUPRC of 0.855. At the 0.5 confidence threshold cut-off, the precision was 81.0%, the recall was 77.1% and F1 score was 0.79. The mean calculated accuracy for all phases was 96.0%.

Table 1 shows the overall model and per-phase performance. Precision ranged between 66.7% for capsule polishing and 100.0% for both hydrodissection and lens implantation. Recall ranged from 46.2% and 100% whereas specificity ranged from 94.1 to 100%. The rhexis phase had the lowest sensitivity as well as the lowest F1 score (0.63). The hydrodissection phase had a F1 score of 0.89, the highest across all phases.

**Table 1:** Performance and evaluation of the overall model and by phase.

	Number	TP	FP	TN	FN	AUPRC	PPV	Sensitivity	Specificity	F1 score
Overall	144	NR	NR	NR	NR	0.855	81.0%	77.1%	98.0%	0.79
Incision	12	10	3	129	2	NR	76.9%	83.3%	97.7%	0.80
Viscous agent injection	26	21	7	111	5	NR	75.0%	80.8%	94.1%	0.79
Rhexis	13	6	0	131	7	NR	100.0%	46.2%	100.0%	0.63
Hydrodissection	12	12	3	129	0	NR	80.0%	100.0%	97.7%	0.89
Phacoemulsification	13	12	3	128	1	NR	80.0%	92.3%	97.7%	0.86
Irrigation & aspiration	16	11	3	125	5	NR	78.6%	68.8%	97.7%	0.73
Capsule polishing	14	10	5	125	4	NR	66.7%	71.4%	96.2%	0.68
Lens implantation	12	9	0	132	3	NR	100.0%	75.0%	100.0%	0.86
Viscous agent removal	13	9	1	130	4	NR	90.0%	69.2%	99.2%	0.78
Tonifying & antibiotics	13	11	0	131	2	NR	91.7%	84.6%	100.0%	0.88

*TP* True positive, *FP* False positive, *TN* True negative, *FN* False negative, *AUPRC* Area under the precision-recall curve, *PPV* Positive predictive value.

Table 2 presents the percentage of correct prediction and the most common misclassifications per phase. The model accurately classified hydrodissection video segments 100% of the time. Its lowest performance was with the rhexis phase, being able to correctly identify only half of the video segments (53.85%).

**Table 2:** Misclassification matrix.

True label	Correct prediction	Misclassification		
		Most common	2^nd^ most common	3^rd^ most common
Incision	91.67%	Viscous inj. (8.3%)	–	–
Viscous agent injection	80.77%	Incision (7.69%)	Capsule polishing (7.69%)	Hydrodissection (3.85%)
Rhexis	53.85%	Viscous injection (30.77%)	Hydrodissection (7.69%)	Phacoemulsification (7.69%)
Hydrodissection	100.00%	–	–	–
Phacoemulsification	92.31%	Hydrodissection (7.69%)	–	–
Irrigation & aspiration	68.75%	Capsule polishing (12.5%)	Phacoemulsification (12.5%)	Viscous removal (6.25%)
Capsule polishing	71.43%	Viscous injection (14.29%)	Incision (7.14%)	Phacoemulsification (7.14%)
Lens implantation	75.00%	Tonifying (16.67%)	Viscous injection (8.33%)	–
Viscous agent removal	76.92%	Irrigation (23.08%)	–	–
Tonifying & antibiotics	84.62%	Capsule polishing (15.38%)	–	–

Table 3 displays the overall model and per-phase performance of the model when tested on the CATARACTS dataset (external validation). Average precision was 54.2%, ranging from 0.0% for irrigation and aspiration to 90.0% for hydrodissection. Recall was 61.1% (ranging between 0.0% and 100.0%) and specificity was 96.2% (ranging between 91.0% and 99.0%). Average accuracy was 93.0%.

**Table 3:** External validation of the overall model and by phase.

	Number	Proportion	TP	FP	TN	FN	PPV	Sensitivity	Specificity	Accuracy
Overall	100	100.0%	NR	NR	NR	NR	54.2%	61.1%	96.2%	93.0%
Incision	10	10.0%	8	5	95	2	61.5%	80.0%	95.0%	93.6%
Viscous agent injection	20	20.0%	10	9	91	0	52.6%	100.0%	91.0%	91.8%
Rhexis	10	10.0%	1	4	96	9	20.0%	10.0%	96.0%	88.2%
Hydrodissection	10	10.0%	7	5	95	3	58.3%	70.0%	95.0%	92.7%
Phacoemulsification	10	10.0%	9	1	99	1	90.0%	90.0%	99.0%	98.2%
Irrigation & aspiration	10	10.0%	0	1	99	10	0.0%	0.0%	99.0%	90.0%
Capsule polishing	0	0.0%	0	0	100	0	NR	NR	NR	NR
Lens implantation	10	10.0%	2	1	99	8	66.7%	20.0%	99.0%	91.8%
Viscous agent removal	10	10.0%	9	4	96	1	69.2%	90.0%	96.0%	95.5%
Tonifying & antibiotics	10	10.0%	9	4	96	1	69.2%	90.0%	96.0%	95.5%

*TP* True positive, *FP* False positive, *TN* True negative, *FN* False negative, *PPV* Positive predictive value.

## Discussion

In this study, we evaluated the accuracy of Google AutoML Video Intelligence at classifying cataract surgery phases. This model was developed by physicians with no coding experience using two publicly available datasets. The use of AutoML in medical imaging has been proven to be effective, being comparable to bespoke DL models. Korot et al. recently published a study where they evaluated the performance of various AutoML platforms at classifying fundus photography and OCT. Some of these platforms had a mean F1 score above 0.90^9^. Despite that, no studies have looked at the use of AutoML for medical video classification and to our knowledge, this is the first assessment of the performance of a DL model produced by AutoML in surgical video classification. We can foresee a wide variety of applications for AutoML video classification in ophthalmology such as vitreoretinal surgery phase classification and even nystagmus identification.

Our model had an AUPRC of 0.855, a precision of 81.0%, a recall of 77.2% and an accuracy of 96.0%. Previously published bespoke DL cataract surgery classifications have examined cataract surgery instrument detection as well as surgical phase detection. Primus et al., who published the Cataract-21 dataset, evaluated the performance of a CNN to assign video frames to one of ten surgery phases^17^. The mean F1 score of 0.68, which could be improved to 0.75 when inputting temporal information (i.e. starting time of the video frames) Our model had a higher F1 score (0.79 vs 0.75), PPV (81.0% vs 74.0%) and higher sensitivity (77.1% vs 72.0%) without using temporal information. Zisimopoulos et al.used segmented phase boundaries and assigned phase labels for cataract surgery^18^. They used a public dataset containing 50 videos with a model classifying video segments among 14 different phases. Although our datasets differed, our AutoML platform revealed higher accuracy (96.0% vs. 78.3%) and F1 score (0.79 vs. 0.75) than their bespoke model. Yu et al.explored five different DL algorithms for cataract surgery phase detection^3^. Three used instrument labels and two used only images (model 3 and 4). Because the authors reported sensitivity, specificity and precision for each phase, we calculated the unweighted average of these parameters to obtain an estimate of the overall performance of the algorithm. The two models had an accuracy of 95.6% and 92.1% respectively. Those models were the most accurate algorithms that we identified through our literature search, and our AutoML model provided comparable performance (96.0%). The state of the art in cataract surgery classification is summarized in Table 4.

**Table 4:** Summary of cataract phase classification models.

	Dataset name	Dataset size	Method	AUPRC	AUROC	PPV	Sensitivity	Specificity	Accuracy	F1 score
Touma et al. (this study)	Cataract 21 & Cataract 101	122	Google AutoML Video Intelligence	0.855	NR	81.0%	77.1%	98.0%	96.0%	0.79
**Yu et al.**^3^										
Model #3	Own dataset	100	CNN input with cross-sectional image data	NR	0.712	78.6%	74.5%	97.5%	95.6%	NR
Model #4	Own dataset	100	CNN-RNN input with a time series of images	NR	0.752	62.0%	59.3%	95.6%	92.1%	NR
Primus et al.^19^	Cataract 21	21	GoogLeNet CNN	NR	NR	69.0% (74.0%)	67.0% (72.0%)	NR	NR	0.68 (0.73)
Zisimopoulos et al.^20^	CATARACTS	50	ResNET-RNN	NR	NR	NR	NR	NR	78.3%	0.75
Quellec et al.^21^	Own dataset	186	Adaptive spatiotemporal polynomial (real-time detection)	NR	NR	NR	NR	NR	85.3%	NR
Qi et al.^22^	Cataract 101	101	ResNET (real-time detection)	NR	NR	NR	NR	NR	88.1%	NR
Charrière et al.^23^	Own dataset	30	Bayesian network (real-time detection)	NR	0.828	NR	NR	NR	NR	NR
Lalys et al.^24^	Own dataset	20	Dynamic time wrapping and hidden Markov models (real-time detection)	NR	NR	NR	NR	NR	95.0%	NR

*AUPRC* Area under the precision-recall curve, *AUROC* Area under the receiver operating characteristic, *PPV* Positive predictive value.

Moreover, some models were developed to be able to identify surgical phases in real-time. Quellec et al. produced an algorithm able to segment a video in real-time between idle phases (no relevant motion is visible) and action phases. When an idle phase was detected, the previous action phase was categorized, and the following action phase was predicted. The real-time segmentation reached an accuracy of 79.3%, and it could be increased to 85.3% once the entire surgery was available^19^. Lalys et al. proposed a method where six binary visual clues were automatically extracted and served as input to an algorithm using dynamic time warping and hidden Markov models. Their model achieved a computation time of approximately 3 s^22^. In comparison, our model uses pre-segmented videos and can only be used asynchronously.

The AutoML model performed well across most surgical phases but some disparities occurred. The lowest performances were for the rhexis, capsule polishing and irrigation/ aspiration phases. Rhexis and capsule polishing were most commonly misclassified as viscous agent injection. One plausible explanation for this discrepancy could be that both viscous injection and capsule polishing use a 27G cannula in this dataset. Thus, it is possible that the model learned to detect certain instruments and infer the phase based on which instrument was detected. The rhexis also involved a long and thin instrument entering the eye from the main incision, which could be misclassified as a 27G cannula. Another potential confounder for the model is phase length; it is possible that phases with shorter length were confused with one another more commonly than with long phases. Irrigation and aspiration was most commonly mistaken for capsule polishing and phacoemulsification. The confusion with phacoemulsification could stem from the presence of similar instruments in the eye (i.e., large instruments) going through the main incision. One way to improve performance would be to have access to saliency maps (or pixel attribution) that allows the users to see which pixels of the images/videos contributes the most to the algorithm’s predictions. This provides some interpretability and understanding of the algorithm’s decision-making process. It allows users to identify patterns that could improve performance and generalizability. This is however not presently accessible in the Google Cloud AutoML Video Intelligence platform.

The model had a lower performance when used on the CATARACTS dataset. There are several technical reasons that can explain the decrease in performance. First, the tools used between the two datasets were slightly different in appearance. For instance, the irrigation and aspiration handpiece has a more pronounced angled tip in the Cataract 21 + 101 datasets than the one in the CATARACTS dataset. Second, the way the various surgical steps are performed varies from one dataset to the other. For example, in the rhexis phase, the capsulorhexis forceps were used in the Cataract 21 + 101 datasets, whereas in the CATARACTS dataset, the capsulorhexis cystotome was mainly employed. Also, in the irrigation and aspiration phase, the micromanipulator is used by the surgeons in the Cataract 21 + 101 datasets, but not by those performing the CATARACTS surgeries. Third, the image quality and color are significantly different between the two datasets, which could explain the difficulty for the model when used on the second dataset. Finally, the discrepancy between the phases’ length (average of 8 min 5 s versus 9 min 24 s per surgery) could partially explain the drop in performance.

AutoML algorithms can have multiple academic applications. Models that can automatically segment and classify surgical videos could allow the creation of extensive libraries containing video segments that could be filtered according to the phase. Residents and ophthalmologists could watch these videos to improve their performance if that specific segment is an area that requires improvement. Another eventual application would be real time identification of surgery phases. Such algorithms could be used in the operating theater to automatically recognize surgical complications or adjust the lighting to better highlight the red reflex. Our current model is limited by the fact that it cannot automatically split the surgical video into segments, but once this hurdle is overcome, AutoML algorithms would be equal to bespoke model in terms of clinical, educational and academical usefulness.

One of the strengths of this study is that our results can be reproduced by other groups given the use of public datasets and the available free trial of Google AutoML Video Intelligence. By combining two public datasets, we were able to have more than 100 video segments per phase which leads to better model training. A paper by Hanussek et al. compared various AutoML models to hand-designed models and concluded that in general, outperformance by AutoML frameworks was dependent on the underlying dataset or task type rather than the choice of algorithm^25^. We believe that this was the case with our model as we had the biggest dataset compared to the other studies discussed in this paper, allowing us to achieve a better performance. Also, the Google AutoML platform provided multiple performance metrics that allowed us to analyse the overall algorithm performance, as well as phase-specific performance. This helped us identify some of the shortcomings of the model and dataset. Producing such an algorithm using AutoML can be done in only a few hours once the data is collected and correctly labelled. This is an important advantage over handcrafted models that require extensive production time. The web user-interface is intuitive with good documentation explaining the different steps, allowing non-experts to quickly get familiar with the concepts and usage of the platform.

This study has a few limitations. First, when the model was tested on a different dataset, its discriminative performance was lower, indicating limited generalizability at this current stage. Model training using varied datasets from different surgeons who use different techniques can improve the model. Also, the model was able to accurately assign labels to pre-segmented surgery videos, but for real-life application, the algorithm needs to automatically segment the video and assign phase labels. A limiting factor of the implementation of AutoML models in the clinical setting is their black-box nature. Being able to recognize which hyperparameters, architectures or methods lead to better performance is an asset of manually produced models.

In summary, we demonstrated the feasibility of producing deep learning models by ophthalmology trainees without coding experience through the use of AutoML. Our model was better performing than bespoke models derived by AI experts. This represents a step forward towards the democratisation of artificial intelligence and its multiple applications. Researchers and clinicians who do not have sufficient knowledge in programming and data science are able to use these platforms to produce robust algorithms that can help them in their projects and clinical decisions. Ultimately, the translation of these technologies into successful and meaningful clinical applications will require collaboration between clinicians and DL experts to avoid biased results and offer a better understanding of the underlying processes.

## Supplementary Information

Supplementary Information.
