# Supplementary material for: Development of a code-free machine learning model for the classification of cataract surgery phases
Source: Sci Rep. 2022 Feb 14;12:2398. doi: 10.1038/s41598-022-06127-5 (PMC8844421; doi:10.1038/s41598-022-06127-5)
Supplement: Supplementary file 1 — Supplementary Information. [file 41598_2022_6127_MOESM1_ESM.docx]

Supplementary Material

**Development of a Code-Free Machine Learning Model for the Classification of Cataract Surgery Phases**

Samir Touma^1,2^, Fares Antaki^1,2^, Renaud Duval^1,2^

1. Department of Ophthalmology, Université de Montréal, Montréal, Québec, Canada
2. Centre Universitaire d’Ophtalmologie (CUO), Hôpital Maisonneuve-Rosemont, CIUSSS de l’Est-de-l’Île-de-Montréal, Montréal, Québec, Canada

**Corresponding author:** Renaud Duval, MD, CM; Centre Universitaire d’Ophtalmologie (CUO), Hôpital Maisonneuve-Rosemont, CIUSSS de l’Est-de-l’Île-de-Montréal, 5415 boulevard de l'Assomption, Montréal, Québec, Canada, H1T 2M4. Email: renaud.duval@gmail.com

|  | | | | |
| --- | --- | --- | --- | --- |
| **Surgery Phase** | **Training Data** | **Test Data** | **Total** | **Distribution** |
| Incision | 113 | 12 | 125 | **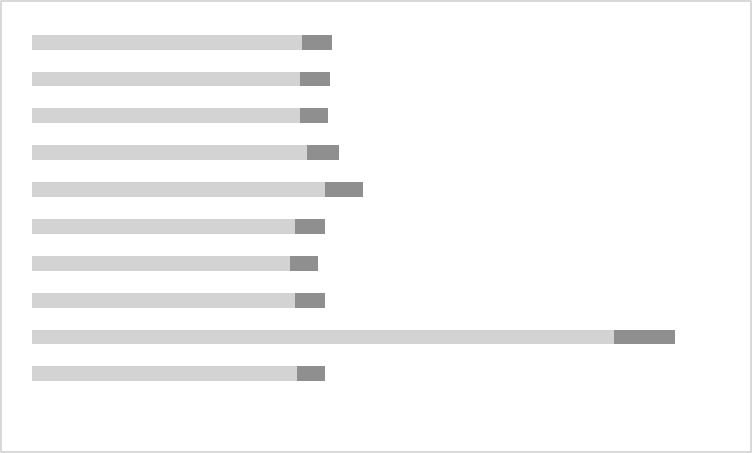** |
| Viscous agent injection | 248 | 26 | 274 |  |
| Rhexis | 112 | 13 | 125 |  |
| Hydrodissection | 110 | 12 | 122 |  |
| Phacoemulsification | 112 | 13 | 125 |  |
| Irrigation and aspiration | 125 | 16 | 141 |  |
| Capsule polishing | 117 | 14 | 131 |  |
| Lens implantation | 114 | 12 | 126 |  |
| Viscous agent removal | 114 | 13 | 127 |  |
| Tonifying and antibiotics | 115 | 13 | 128 |  |

**Supplementary Table 1. Cataract surgery phases distribution.** Shows the number of instances of each phase and the distribution across training and testing. The median number of videos per phase was 114 for the training data and 13 for the testing data.
Under the distribution column, we can see in dark grey the proportion of videos used for testing compared to the ones used for training in light grey.


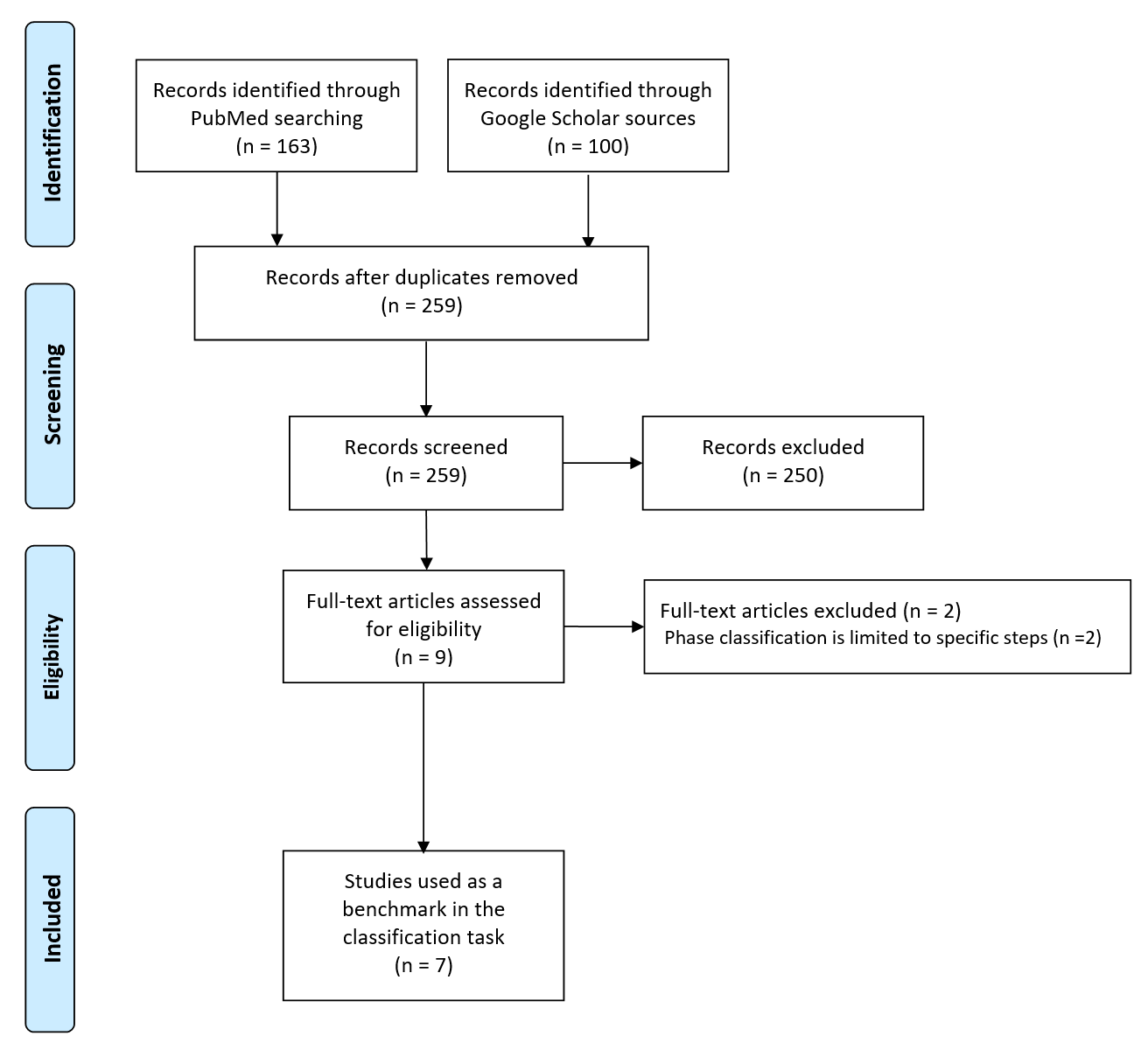


**Supplementary Figure 1. Data flowchart for the literature review**

The MEDLINE (via PubMed) search strategy was the following: (artificial intelligence OR deep learning OR machine learning) AND ((cataract surgery [MeSH Major Topic]) OR (cataract surgery phase classification) OR (cataract surgery phase recognition) OR (automated phase identification). All references from PubMed were imported.

The Google Scholar search strategy was: (artificial intelligence OR deep learning OR machine learning) AND (((cataract surgery) OR (cataract surgery phase classification)) OR (cataract surgery phase recognition) OR (Automated Identification Phases)). The first 100 results from Google Scholar were imported.
